# Fully Microfabricated Surface Acoustic Wave Tweezer for Collection of Submicron Particles and Human Blood Cells

**DOI:** 10.1021/acsami.3c00537

**Published:** 2023-05-15

**Authors:** Armaghan Fakhfouri, Melanie Colditz, Citsabehsan Devendran, Kateryna Ivanova, Stefan Jacob, Adrian Neild, Andreas Winkler

**Affiliations:** † Leibniz-IFW Dresden, Helmholtzstr. 20, 01069 Dresden, Germany; ‡ Department of Mechanical and Aerospace Engineering Monash University, Clayton, Victoria 3800, Australia; ¶ Physikalisch-Technische Bundesanstalt, Bundesallee 100, 38116, Brunswick, Germany

**Keywords:** Surface acoustic waves, acoustic tweezers, acoustofluidics, cell manipulation, nanoparticle concentration

## Abstract

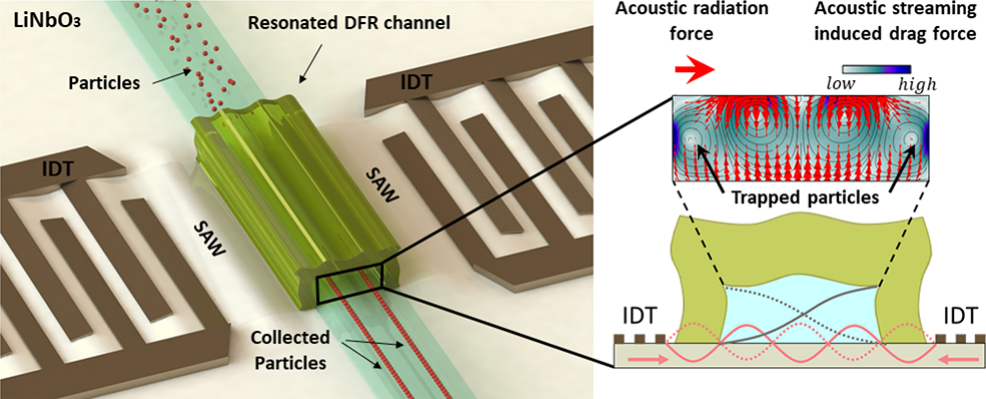

Precise manipulation of (sub)micron particles is key for the preparation, enrichment, and quality control in many biomedical applications. Surface acoustic waves (SAW) hold tremendous promise for manipulation of (bio)particles at the micron to nanoscale ranges. In commonly used SAW tweezers, particle manipulation relies on the direct acoustic radiation effect whose superior performance fades rapidly when progressing from micron to nanoscale particles due to the increasing dominance of a second order mechanism, termed acoustic streaming. Through reproducible and high-precision realization of stiff microchannels to reliably actuate the microchannel cross-section, here we introduce an approach that allows the otherwise competing acoustic streaming to complement the acoustic radiation effect. The synergetic effect of both mechanisms markedly enhances the manipulation of nanoparticles, down to 200 nm particles, even at relatively large wavelength (300 μm). Besides spherical particles ranging from 0.1 to 3 μm, we show collections of cells mixed with different sizes and shapes inherently existing in blood including erythrocytes, leukocytes, and thrombocytes.

## Introduction

Surface acoustic wave (SAW) based acoustic tweezers are an emerging technology for precise, selective, and contactless manipulation of microscale objects in diverse applications ranging from surface cleaning,^1,2^ sensing,^3^ and microrobotics,^4^ to tissue engineering,^5,6^ drug delivery,^7^ and personalized diagnostics.^8−10^ SAW tweezers owe their increasing popularity, particularly for biologically oriented applications, to the substantive yet biocompatible acoustic forces enabling a robust manipulation entirely based on the bioparticles intrinsic properties while sustaining the morphology and viability of biological samples.^11−13^

Typical SAW tweezers rely on acoustic radiation force (*F*_Rad_), an effect that directly acts on the particles suspended within an acoustic field, as the driving mechanism for controllable particle manipulation,^14^ separation,^15,16^ patterning,^17,18^ and filtration.^19^ Besides acoustic radiation, the propagation of acoustic energy into a viscous fluid gives rise to a secondary effect, termed acoustic streaming, which generates localized steady flow and, in turn, exerts a drag force (*F*_d–streaming_) on the immersed particles. In most well-established SAW-based acoustic tweezers, the influence of acoustic radiation force is progressively hindered by acoustic streaming induced drag force as the particle dimension reduces. The swirling motion of streaming typically disrupts the ability of radiation forces to effectively collect submicron particles.^20^ Therefore, and despite the growing adaptation of SAW systems for micromanipulation, the focusing capability of the technology is limited as particles progress to submicron scales.

A number of strategies have been employed to try to address submicron-scale manipulation in SAW systems. One approach is to use secondary Bjerknes force, a type of radiation force between particles, to trap submicron particles against a flow onto vibrating seed particles.^21−23^ Others have adopted different methods, aimed at retaining the possibility of continuous flow processing, such as pronouncing the streaming field and using the presence of points of purely rotational flow structures for the concentration of submicron particles.^24,25^ Streaming-induced manipulation alone, however, lacks the high size selectivity required for many diagnostic and therapeutic purposes. Other techniques seek to increase the magnitude of primary acoustic radiation force, by reducing the acoustic wavelength to scales comparable with submicron and nanometer particles.^26,27^ Although these approaches can effectively overcome a certain degree of mismatch between radiation force dominance and submicron scale manipulation, increasing the primary or secondary radiation force, sufficiently to impact submicron particles, is realized at a cost of added complications (e.g., drastically reduced volume flow and increased clogging in smaller channels) that may restrict the practical use of such techniques for real-life applications.

There are two main acoustic excitation mechanisms in microfluidics including bulk acoustic waves (BAW) and surface acoustic waves (SAW). In BAW approaches, by resonating the entire fluid-filled microchannel using a piezoelectric transducer adhered to the rigid channel wall, acoustic waves are excited across the fluid volume (Figure S1, A).^28,29^ SAWs, however, are excited only on a part of the surface of a piezoelectric substrate using interdigital transducers (IDTs) (Figure S1, B).^30^ They can readily localize and orient acoustic fields in specific, desired regions of a microchannel and are suited for cost-efficient integration into a disposable fluidic chip. This imparts great advantages on BAW systems which have high throughput but limited functionality.

Besides actuation method, the microfluidic channel, containing the fluid medium, has a critical role in the patterns of the acoustic field and the resultant acoustic force distribution across the fluid. Current SAW tweezers often employ polymeric channels, such as polydimethylsiloxane (PDMS), which are capable of attenuating most of the acoustic energy owing to their similar acoustic impedance compared to the liquid (i.e., water) in contact. In general, the high-absorption of acoustic energy by the channel wall and ceiling material minimizes the reflections at the channel/liquid intersections permitting certain possibilities such as particle manipulation using traveling field,^31^ pulsed field,^32^ focused field,^33,34^ and the SAW driven diffraction features.^35,36^ The manual handling involved in the fabrication and placement of such channels, however, not only reduces their reproducibility but renders them unsuitable for applications that require precise placement of channel features relative to the features of the SAW wavefield. Here, we demonstrate the straightforward development of lithographically defined microfluidic channels out of laminated dry film resist (DFR). Through the high-resolution realization of the channel features with the exact intended dimensions, architecture, and placement, the DFR-based approach allows for the fabrication of highly reproducible microchannels.

While substantial progress has been made in understanding the actuation and development of the SAW-driven acoustic effects across the liquid contained in the “absorbent” channel, very little attention has been devoted to considering the actuation of the liquid bound within the “reflective” channels. SU-8 photoresist offers a promising alternative as a material for reflective microchannels, and in previous work, we have demonstrated successful use of SU-8 as a michrochannel wall material for SAW-driven aerosol generation.^37^ However, the length of such a channel is ultimately limited to only a few mm, due to diffusion limitation of developer and reaction products. Development of a several cm long, fully enclosed microchannel with SU-8 seems not possible with the current fabrication technologies. Using SU-8 for channel walls and another elastic material (e.g., PDMS) as a cover allows a precise lithographic arrangement of the channel walls and avoids PDMS soft-lithography.^38^ Excitation of the whole channel resonance, however, is only possible in enclosed microchannels with “acoustically-hard” enclosing walls, including the cover.

The use of a fully reflective channel in a SAW tweezer has only recently been realized in glass microchannels adhered to the piezoelectric substrate via an adhesive layer.^39−42^ In those cases, SAW is reported to give rise to strong channel vibrations and even to excitation of the whole-channel resonance if the system is designed accordingly as shown in Figure S1, C. SAW-driven channel resonance uniquely allows for tuning the acoustic streaming induced force and acoustic radiation force to complement, rather than compete or dominate, each other toward collecting submicron particles. On this evidence, we argue that to reduce the size of the collectable particles and achieve optimum manipulation resolution of SAW-tweezers there needs to be a rethink of microchannel material and the fabrication approach. While glass is highly reflective for the effective creation of channel resonance, the availability of geometry and design of glass microchannels for the excitation of higher-order vibrational modes is undesirably limited. In addition, the fabrication of glass-based SAW tweezers, with a high sensitivity of the resonance mode to dimensions and features of the microchannel, suffers from several drawbacks regarding reproducibility, suitability for large-scale automated production, and versatility owing to the complicated and costly structuring of glass along with the need for precise placement on the chip.

**Figure 1:**
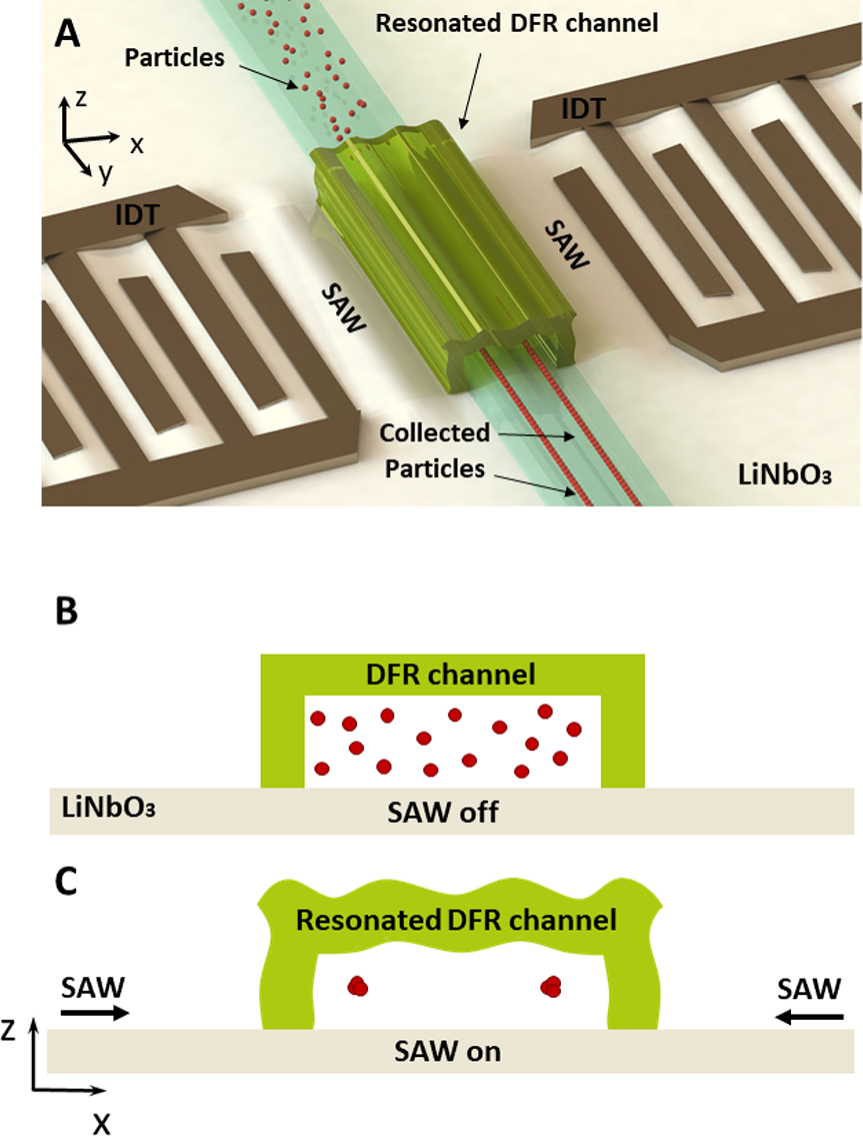
Principle of SAW-driven channel resonance. (A) Reproducible and high-precision definition of stiff microchannel can be integrated onto SAW tweezers, by means of laminated DFR fabrication approach, to reliably actuate the entire microchannel cross-section giving rise to the synergetic effect of *F*_Rad_ and *F*_d–streaming_ for effective collection and focusing of particles regardless of their size and shape. Cross-sectional view of the microfluidic channel when (B) SAW is off and particles occupy random positions across the channel and (C) SAW is applied driving whole channel resonance (exaggerated vibration depicted), thereby collecting particles at the equilibrium positions where both acoustic forces, *F*_Rad_ and *F*_d–streaming_, are minimum.

Here, we report that the use of DFR lamination, a commonly used approach for MEMS fabrication and wafer-level packaging,^43,44^ enables the manufacturing of microchannels with precise dimensions and detailed architecture to reliably actuate the intended whole-channel resonance. The high-precision realization of microchannels promoted by the DFR-based approach permits the practical application of finite element analysis (FEA) numerical predictions to real-life devices. Herein, a desirable channel movement identified numerically can successfully localize the acoustic streaming field at favorable locations while minimizing it across the rest of the microchannel. Using the adjusted fields, this method enables the formation of acoustic streaming-induced drag force to complement, rather than compete or dominate, the acoustic radiation force and act as an assisting mechanism to efficiently collect submicron particles, down to 200 nm particles, and form two highly focused particle streams inside a 150-μm-wide and 50-μm-high channel at the wavelength of 300 μm as shown in Figure 1. While a popular approach in MEMS, circuit board fabrication, and, only recently, for passive microfluidics,^45−47^ to the best of our knowledge, no report of using laminated DFR for particle manipulation purposes in SAW-driven technologies has been proposed. The defined and reproducible manufacture of SAW acoustofluidic chips on the wafer scale using this technique obviates the fundamental limits of soft-lithography including bonding inconsistencies, alignment issues, and microchannel deformation. The production and usage of these chips for high-throughput micro- and nanoscale particle concentration, shown in this work, proved the technology to be a straightforward solution for the translation of SAW acoustofluidics toward large-scale industrial implementation and biomedical studies.

## Results

**Figure 2:**
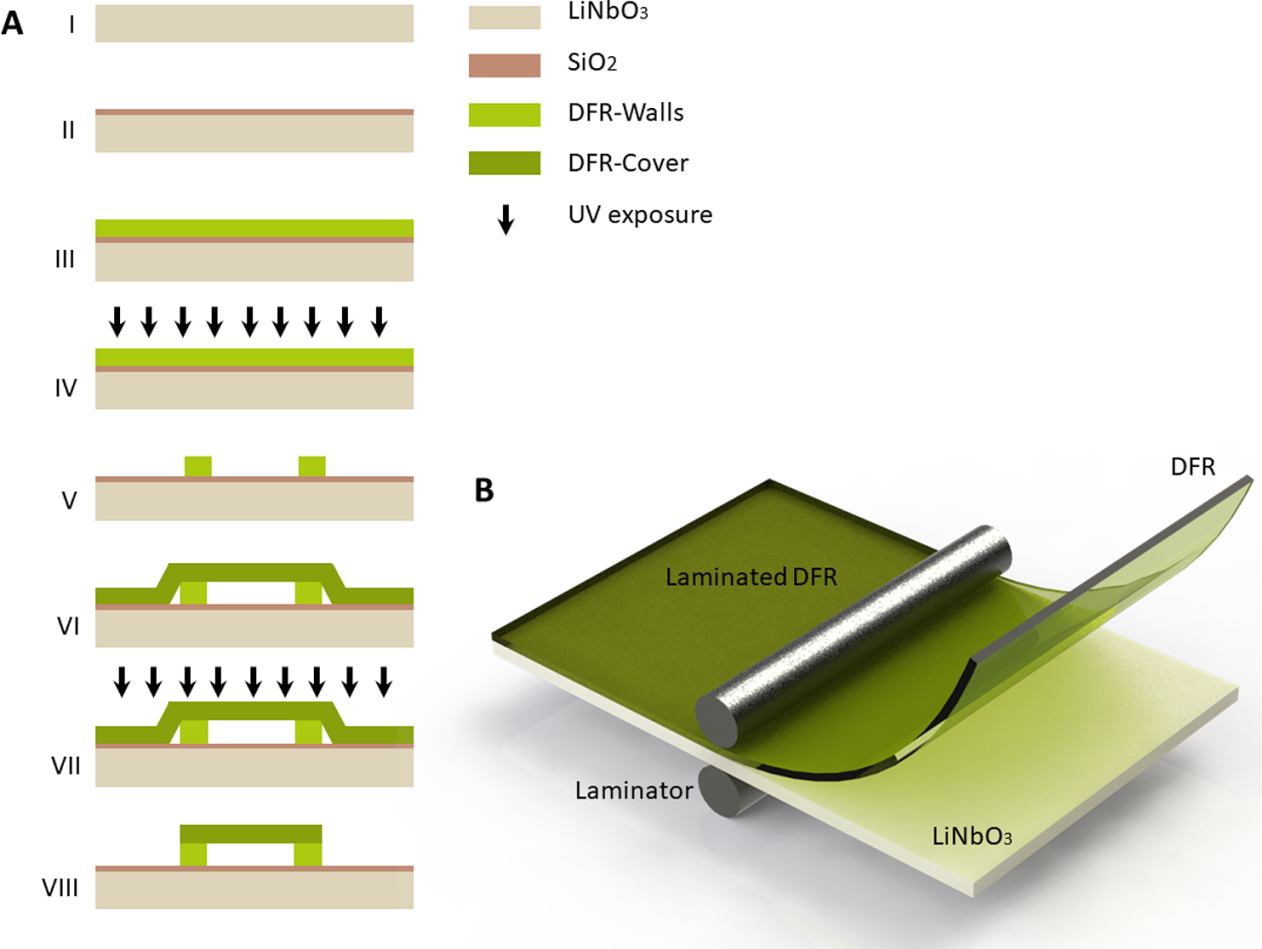
Fabrication procedure of laminated-DFR μchannel. (A) Fabrication process diagram where (A-I) LiNbO_3_ substrate (A-II) is deposited with SiO_2_, then epoxy-modified (A-III) and (B) DF-3500 DFR is laminated on the substrate, (A-IV) then exposed to the ultraviolet light using a maskless laser-based technique. (A-V) Microchannel walls are developed and heat treated. (A-VI) Another layer of DF-3500-DFR is laminated, (A-VII) then exposed to the ultraviolet light through the maskless laser-based technique, and (A-VIII) microchannel cover is developed and heat treated.

**Figure 3:**
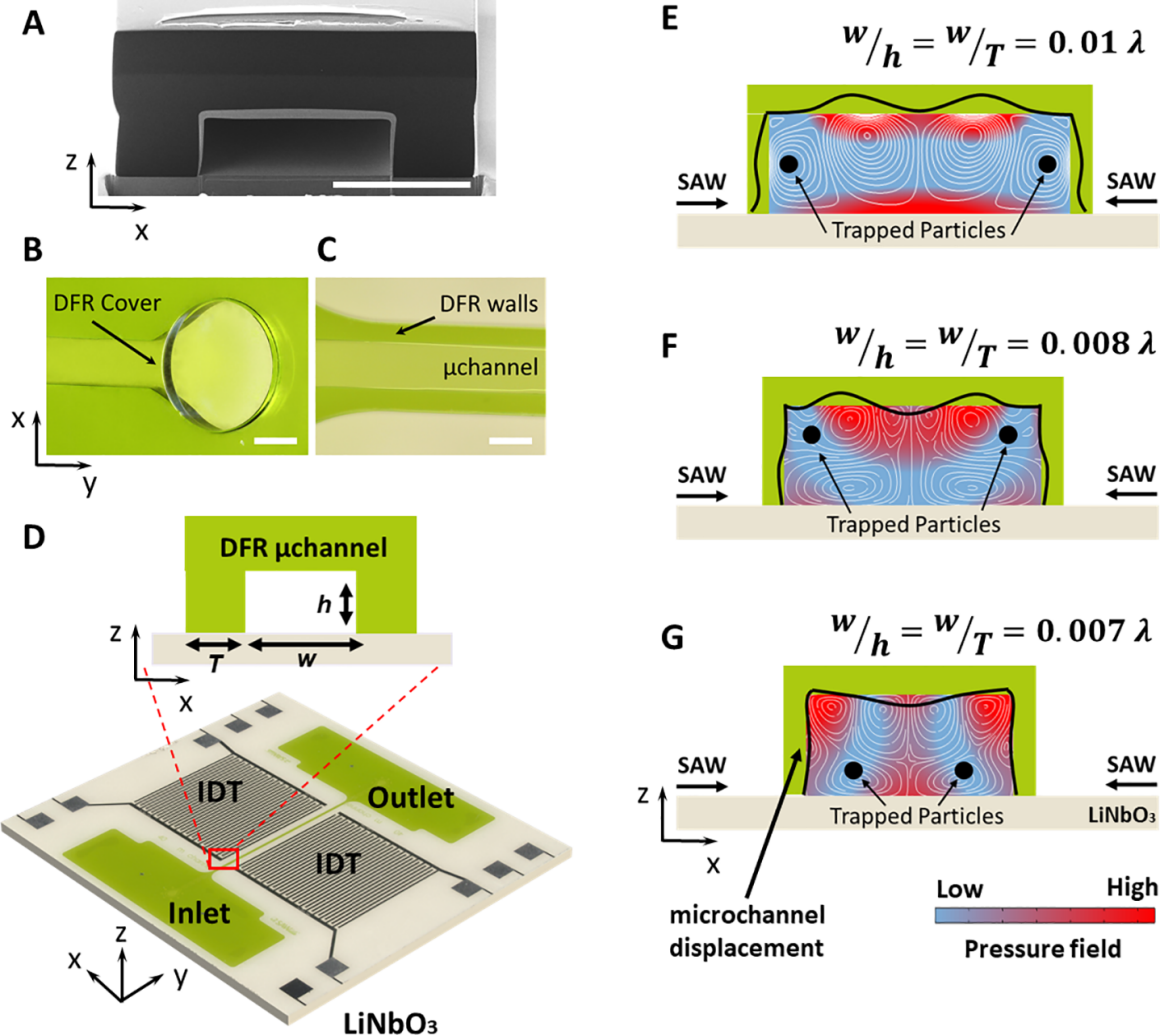
Principle, parameters, and mode shape of realized SAW-driven DFR microchannel resonance setup. (A) Ion-beam cut electron microscopy of microchannel cross-section (light gray material deposited for imaging purposes). (B and C) Optical images of the microchannel walls and cover. (D) Overview of the chip design with a close-up sketch of the microchannel cross-section, where *w* and *h* are microchannel width and height, respectively, and *L* indicates the wall thickness. (E–G) Upon coupling of SAW, a specific microchannel mode shape, predicted numerically, is excited enabling predefined time-averaged acoustic pressure and streaming fields (shown with white lines) cooperating to focus the particles into trapping locations which vary depending on the channel parameters and configurations. Scale bars, (A, B, C) 100 μm.

Inspired by the straightforward DFR lamination-based approaches in MEMS and microfluidics technologies, we present a chip fabrication approach to enhance the reliable replicability of numerical predictions to experimental setup which is the key to achieve whole channel resonance and consequently nanoparticle manipulation. This is possible via uniform and high-precision realization of microchannels (with fabrication accuracy better than 5 μm), according to procedures (A-I) to (A-VIII) as outlined in Figure 2. As can be seen, the initial layer of DFR is laminated on the epoxy-functionalized SiO_2_ (Figure 2, AI-AIII and Figure 2, B) forming a covalent bond and then lithographically defined (Figure 2, A). The second layer of DFR is then laminated and lithographically realized to enclose the microfluidic channel as shown in Figure 3, A to C. As such, the proposed fabrication approach increases the lab-to-real-life translatability of SAW-driven devices via simple, yet efficient and reproducible methods.

Here, the SAW waves, propagating at 12.8 MHz (λ_SAW_ = 300 μm) from a pair of opposing IDTs on a lithium niobate (LiNbO_3_ 128° *YX*) piezoelectric substrate, couple into the microchannel. This leads to resonance along the microchannel cross-section that efficiently couples to the fluid, creating the desired acoustic streaming field. To exhibit the flexibility of DFR-based approach and the strong influence of particle behavior on the choice of channel dimension and architecture, we have optimized three channel designs, through FEA numerical simulations and experimental verification, with channel width to height (w/h) and to wall thickness (w/T) ratios of 0.01λ_SAW_, 0.008λ_SAW_, and 0.007λ_SAW_, as shown in Figure 3, E to G, respectively.

The displacement field, along with the time-averaged absolute acoustic pressure field and the acoustic streaming field were numerically modeled to identify the steady state acoustic radiation force, *F*_Rad_, and the acoustic streaming induced drag force, *F*_d–streaming_, for various particle dimensions. The complementary effect of the acoustic forces (*F*_Rad_ and *F*_d–streaming_) serve as the fundamental mechanism to displace particles from their initial position and eventually capture them in predetermined locations (see Figure 3, E to G) along the microchannel cross-section. Considering that central positioning of particles across the channel height permits higher throughput by reducing the contact possibility of particles/channel-cover and particles/substrate, we have chosen the design where *w*/*h* = *w*/*T* = 0.01 λ_SAW_ (Figure 3E) among the three designs, shown in Figure 3, E to G, for further exploration.

**Figure 4:**
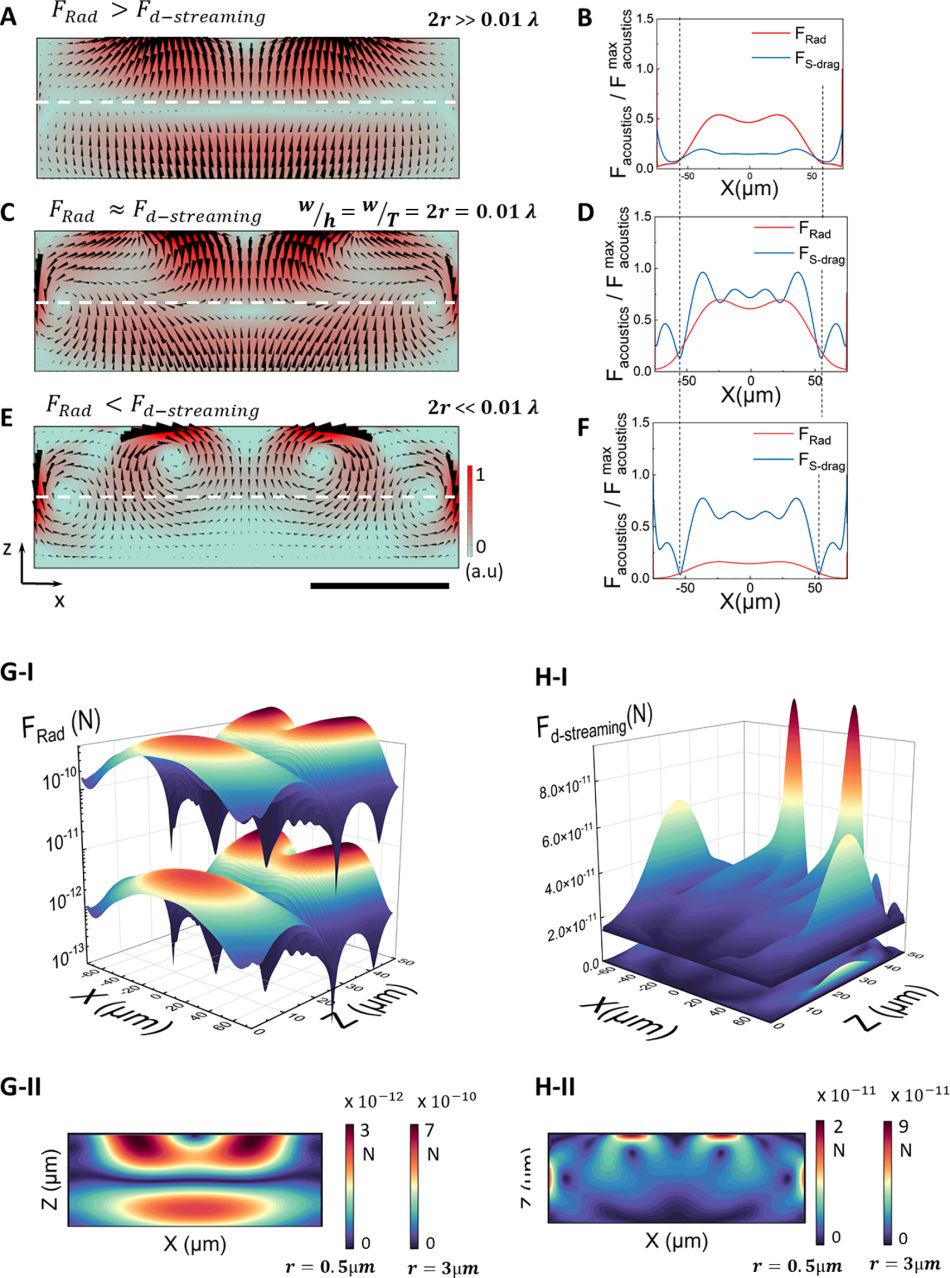
Numerically simulated acoustic radiation force (*F*_Rad_) and acoustic streaming drag force (*F*_d–streaming_). (A–F) Combined effects of both acoustic forces on particles where particle diameter (2*r*) is (A and B) greater than 0.01 λ_SAW_ leading to predominance of *F*_Rad_, (C and D) equal to 0.01 λ_SAW_ with *F*_Rad_ and *F*_d–streaming_ having similar degree of significance, and (E and F) smaller than 0.01 λ_SAW_ resulting in predominance of *F*_d–streaming_. Regardless of dominant initial effect, both forces act together to displace particles and eventually trap them in the locations where both forces are minimum. (G-I) 3D plot and (G-II) top-view of steady-state *F*_Rad_ field, numerically calculated from the time-averaged absolute acoustic pressure field, for 0.5 and 3 μm particles showing the critical role of the particle size in the relative significance of *F*_Rad_. (H-I) 3D plot and (H-II) top-view of the steady-state *F*_d–streaming_ field, numerically calculated from the time-averaged second order velocity field, for 0.5 μm (hidden behind the graph for 3 μm) and 3 μm particles showing the much less significant changes in *F*_d–streaming_ compared to *F*_Rad_ as the particle size varies. The simulated displacement field is adjusted to wavefield measurement results by laser Doppler vibrometry reading 0 to 5 × 10^–4^ μm. Scale bar equals 150 μm (λ_SAW_/2).

Figure 4 shows the overall effect of acoustic force field, consisting of both *F*_Rad_ and *F*_d–streaming_, on particles of different sizes. The final positions of particles, regardless of their size, collide in the steady state. The particle transient displacement profile, however, closely follows the direction of overall force field as shown with black arrows in Figure 4, A, C, E.

Our numerical simulations suggest that for particles with a size 2*r* = 0.01 λ_SAW_, in general, *F*_Rad_ and *F*_d–streaming_ equate in magnitude. In this case and depending on the positioning of a particle in the channel, either force interchangeably could dominate the particle displacement until it is trapped in a position wherein the magnitude of both forces is minimum, e.g., close to the channel edges, as indicated in Figure 4, C and D. Particles larger than 0.01 λ_SAW_ are predominantly manipulated by *F*_Rad_ (Figure 4, A and B), and those smaller than 0.01 λ_SAW_, down to a certain diameter (∼0.0003 λ_SAW_), are mainly affected by *F*_d–streaming_ (Figure 4, E and F) that act, respectively, to move particles from their initial position toward the equilibrium positions. Our observations suggest that the nonlinear effects (e.g., applied power, and hence additional acoustothermal contributions) highly affect particles below 0.0003λ_SAW_, thus their pattern of manipulation and displacement do not follow the physics explained here and require further investigation.

Figure 4, B, D, and F indicate the effect of individual components of overall force field, *F*_Rad_ and *F*_d–streaming_, independent of each other, along the white dashed lines shown in Figure 4, A, C, and E. The final trapping position of particles is indicated with a black dashed line. It can be seen that, the amplitude of streaming induced drag force right at the channel edge is sufficiently large to hinder the radiation force dominated displacement that tend to trap particles at the channel edge, where *F*_Rad_ = 0, even for particles larger than the critical size (2*r* ≫ 0.01λ_SAW_). The amplitude of *F*_d–streaming_ drop to a level comparative to *F*_Rad_ at about 35 μm away from the upper and lower channel edge. Focusing of particle streams at a small distance from the channel edges prevent cell-to-channel adherence avoiding cell damage through cross-contamination and wall-induced shear force. Figure 4, G and H, shows the scaling of absolute values of *F*_Rad_, as a logarithmic function, and *F*_d–streaming_, as a linear function acting on 0.5 and 3 μm particles in a 150-μm-wide channel operating at 12.8 MHz (λ_SAW_ = 300 μm). It can be seen that the change in particle diameter critically affects the magnitude of radiation force (since *F*_Rad_ ∝ *r*^3^),^48^ while it is lightly felt by the streaming induced drag force (since *F*_d–streaming_ ∝ *r*).^49^

**Figure 5:**
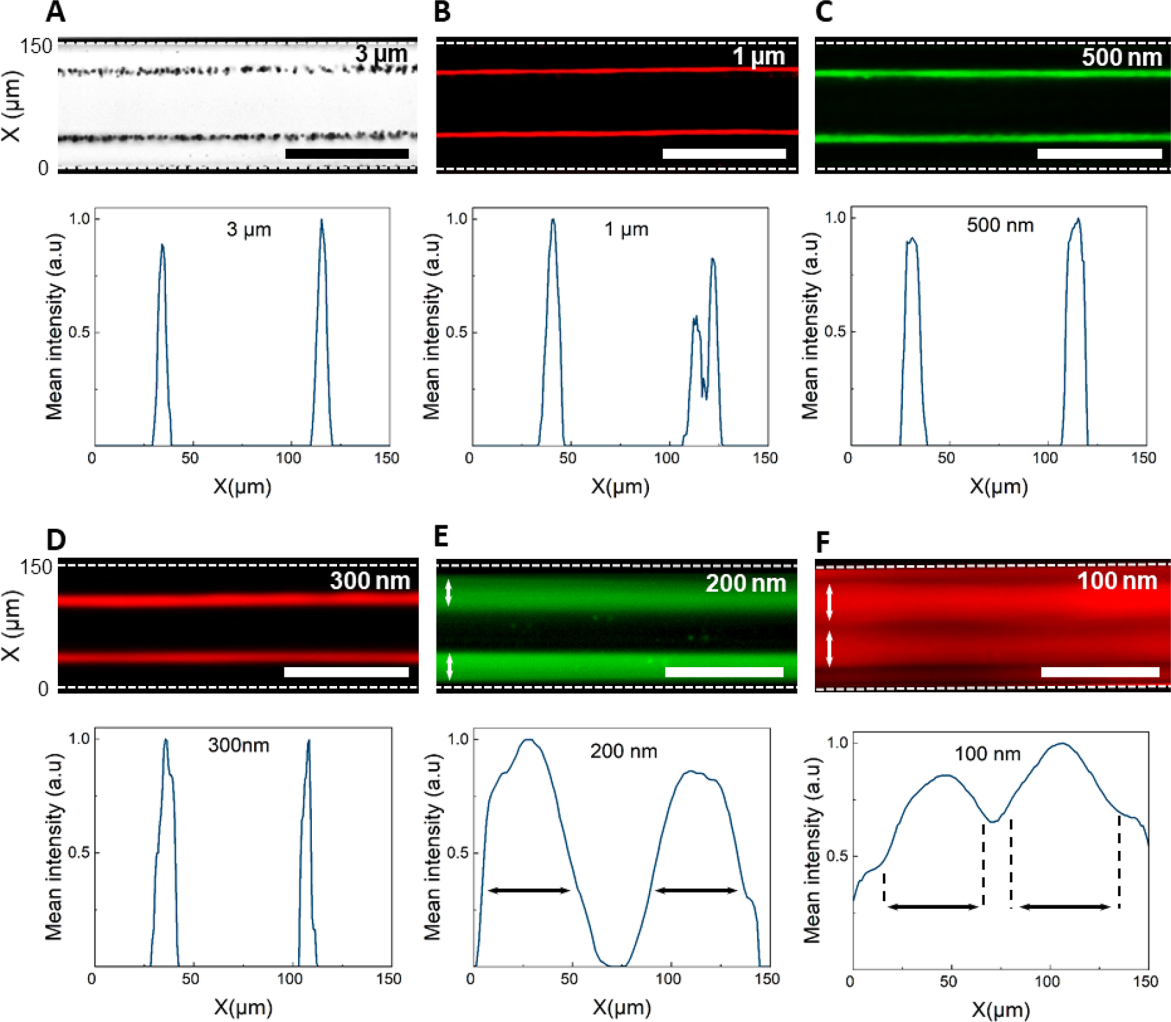
Experimental proof of submicron particle focusing using whole channel resonance using the SAW-driven device described above, with identical straight IDTs (λ_SAW_ = 300 μm, 12.8 MHz) and a channel width of 150 μm (*W* = λ_SAW_/2). We demonstrate focusing at 0.3 μL/min flow rate using polystyrene particles and applied powers of (A) 3 μm and 10 mW, (B) 1 μm and 20 mW, (C) 500 nm and 50 mW, (D) 300 nm and 80 mW, and (E) 200 nm and 600 mW. (F) 100 nm particles are entrained in the acoustic streaming vortices across the whole channel width 700 mW. Scale bars are 200 μm.

The experimental observations (Figure 5) demonstrate the focusing of particles down to 300 nm into two highly focused streams and further down to 200 nm into two slightly broadened streams near the walls of the microchannel. While the verification and the clear differentiation between the dominating mechanisms, either *F*_Rad_ or *F*_d–streaming_, leading to such focused streams are not visually accessible in an experimental setup, the final location of the collected particles closely resembles the numerical predictions. Such locations can be identified through measuring the light intensity distribution across the channel width, as demonstrated in graphs associated with each experimental image in Figure 5, indicating the particle concentration at various locations. Using 300 μm IDTs, operating at 12.8 MHz, Figure 5, A to D, shows highly focused streams of 3 μm, 1 μm, 500 nm, and 300 nm particles at 0.3 μL/min and 10 mW, 20 mW, 60 mW, and 80 mW, respectively, comparing favorably to the FEA simulations, with ±5 μm accuracy of the numerical predictions for the collection positions.

As the particle diameter reduces to 200 nm at 0.3 μL/min applied flow rate and 600 mW applied power, as shown in Figure 5 E, however, the particle streams expand to approximately λ_SAW_/3 of the channel width, separated by a λ_SAW_/3 particle-purified media stream in the center, representing the increased entrainment of particles in the streaming vortices. Such behavior sets the limit for the functionality of the device where the particle streams can be separated from the centered purified liquid, for certain applications. In such a case, though the acoustic forces are insufficient to form highly focused streams. A further reduction of particle diameter to 100 nm, in Figure 5 F, demonstrates a large baseline in the light intensity distribution plot with two weak fluctuations at 0.3 μL/min and 700 mW, indicating behavior dominated by a weak acoustic streaming induced drag force.

**Figure 6:**
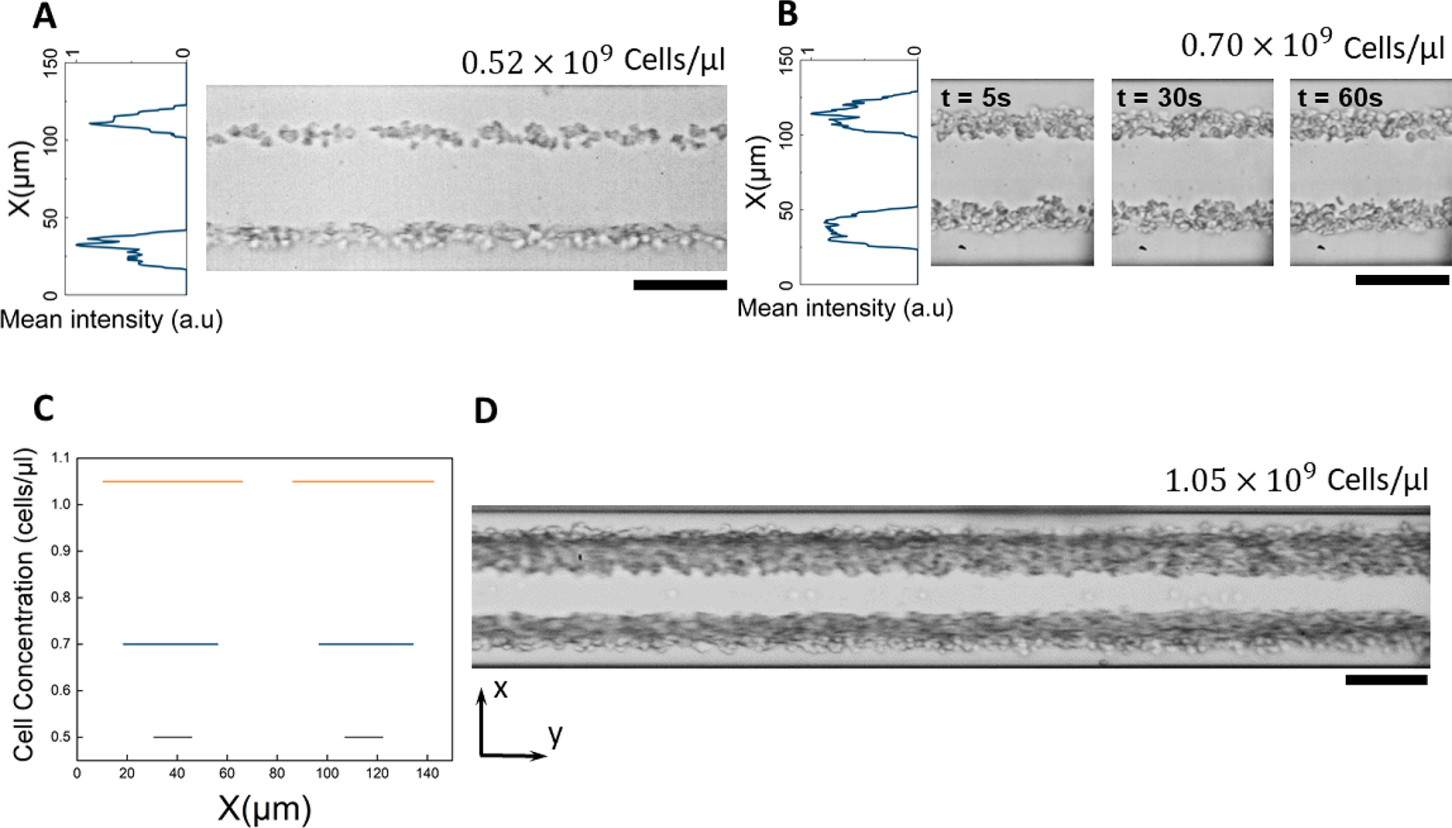
Focusing of human-blood components into two streams. (A) The excitation of SAWs with 12.8 MHz at 400 mW enables focusing of the blood components including erythrocytes, leukocytes, and thrombocytes, with a high flow rate of 25 μL/min, into narrow streams in optical microscopy images and their mean light intensity plots. (B) Cell-focusing occurs immediately and retains a temporal stable stream pattern upon continuous SAW application. (C) Estimation of the mean and standard deviation of the cells focusing location from a total of 20 devices with three cell concentrations shown in (A, B, D). (D) The focused streams of cells are uniform throughout the channel. Scale bars are 75 μm.

The focusing performance in a DFR-microchannel, resonated by SAW, is not restricted to spherical polystyrene particles and can be successfully applied to biological cells. Figure 6 shows simultaneous focusing of different components present in human blood including erythrocytes (7–8 μm), leukocytes (7–20 μm), and thrombocytes (1–4 μm). Here, the human blood is diluted to a concentration of 20% in phosphate buffered saline (PBS) to reduce the initial influence of in-between cell forces, known as Bjerknes force, yet allow for sufficient cell concentration to investigate the sustainability of the technology for a range of cell types and biological particles. While the blood components are larger relative to the focusing resolution of our technology, this clearly demonstrates the device’s capability in handling biological samples with irregular shapes and sizes. Here, using the *w*/*h* = *w*/*T* = 0.01 λ_SAW_ design, with 300 μm wavelength for operation at 12.8 MHz, diluted blood was injected at a high flow rate of 25 μL/min, and all the cellular components, regardless of their size and shape, were effectively focused at a low applied power of 400 mW. The cell capture locations are similar to those of particles; however, the extent of the streams is slightly larger owing to the much higher concentration of cells relative to the particle solutions. Figure 6 B indicates that the cells immediately reach a stable state at which optimized localization occurs and maintain their position in a uniform manner downstream along the microchannel (see Figure 6 D).

As a vital requirement in most of the biological applications, our technology indicates rapid yet stable localization of cells at predetermined positions near the channel walls, leaving purified media in the central region of the channel. We then collect the focused cell streams as well as the purified media (i.e., blood plasma) separately in bifurcated channel outlets for further biological processing, particularly for applications where physical or optical access to plasma is required. In such applications, having two streams instead of one double the capacity to accommodate the cells, reducing cell–cell interaction due to limited space in the microfluidic channels. Figure 6 C indicates the reproducibility of the results regarding focusing of the cell streams in 20 different devices operating at the same performance parameters (i.e., frequency, power, and flow rate). A cell mixture at the concentration of 0.52 × 10^9^ cells/μL, 0.70 × 10^9^ cells/μL, and 1.05 × 10^9^ cells/μL have shown constant focusing behavior where two streams are formed at the averaged location of 37.6 and 117.5 μm. The extend of the focused cell stream increases with the concentration of the cells.

## Discussion

The performance of conventional SAW-driven acoustic tweezers for the purpose of submicron particle focusing and, eventually, their separation can significantly be improved using the DFR-based microchannel approach demonstrated here for the first time. The oscillation of the microchannel cross section to excite the bounded liquid is a common approach in BAW based acoustofluidics, where a piezoelectric chip adhered to a microchannel couple the acoustic waves into the fluid. Typically, an acoustically “hard” microchannel wall material capable of generating strong wave reflections (e.g., silicon or glass) is used in these approaches. The physics underlying BAW channel oscillation has been thoroughly investigated, reporting that the solid–liquid boundary conditions critically influence the formation of resulting acoustic streaming fields across the microchannel.^50,51^

In the system demonstrated here, however, patterns of the channel cover displacement vary to those of the SAW-actuated channel bottom, thus giving rise to an asymmetrical acoustic streaming field where streaming is localized near the DFR-cover/liquid boundary while suppressed near the LiNbO_3_–substrate/liquid boundary as shown in Figure 4 H-II. Through the careful design of the microchannel, this unique feature can be harnessed to establish the otherwise dominant streaming induced drag force to complement the acoustic radiation force for effective collection of submicron particles. In contrast to most SAW-based acoustic tweezers where *F*_d–streaming_ and *F*_Rad_ compete for dominance, we have shown that a favorable combination of such forces, promoted by channel architecture and material properties, eliminates the need for pronunciation of one of these forces against the other. Despite the different localized effects depending on the particle size and location in channel, alignment of the force equilibrium positions, where both *F*_d–streaming_ and *F*_Rad_ are minimum (see Figure 4, A to F) provides a common destination for particle settlement regardless of which acoustic force dominates particle displacement. Such an alignment smoothens the transition from radiation force dominated toward streaming force dominated physics, thereby effectively overcoming the restricted size-dependent ability of typical SAW-devices to focus a much larger size range of target particles.

For microchannel design optimization for a given fluid, constant wavelength, and particle size, the critical parameters include channel width (*w*), channel height (*h*), wall thickness (*T*_w_), and cover thickness (*T*_c_), whereby modification of any individual parameter, independent of the others, change the resultant displacement profile and the corresponding acoustic field (see Figure S2). Our FEA numerical simulations suggest that when *h* = *T*_w_ = *T*_c_ = λ_SAW_/6, the overall phase difference between the outer (air/DFR) and the inner (DFR/liquid) microchannel boundary generate a microchannel oscillation essential for the development of desirable acoustic pressure and acoustic streaming fields. The resultant acoustic forces arising in three channels of differing width, equating to λ_SAW_/3, λ_SAW_/2.5, and λ_SAW_/2, as shown in Figure 3, E to G, has been investigated, verifying the flexibility in choice of the channel dimensions and architecture for synergetic influence of acoustic radiation force and acoustic streaming force for focusing of the particles in (sub)micron size range. While each design offers specific benefits depending on the application, for the experiments shown here, we have chosen the design with λ_SAW_/2 channel width. This design provides the largest spacing between focusing locations across channel width, allowing high fluid throughput, but also minimizes the possibility of particle adherence to the channel cover or to the substrate surface due to centered focusing location across channel height.

As opposed to the conventional device fabrication approaches which lack an appropriate technique for exact replication of the numerically optimized channel parameters and architecture in real-life, introduction of lithographically defined DFR microchannels fabrication to SAW-driven devices not only allows for the exact intended channel geometry and placement of the microchannel with micron resolution (see Figure 2 and Figure 3, A to C) but also enables defined and reproducible device manufacturing on the wafer-scale using typical industrial methods and biocompatible materials, thereby in contrast to soft-lithography, is suited for cost-efficient and mass-production of SAW-acoustofluidic chips. Altogether, design and manufacture of SAW-microchannels from laminated DFR is an elegant, reproducible, and straightforward means, here offering size-independent (down to 200 nm), high throughput (as high as 25 μL/min), rapid manipulation, and efficient pressure-field excitation (with input power as low as 10 mW) and may pave the way to translate SAW tweezers from lab-based proof of concepts to practical larger-scale, life science applications.

We expanded our investigation from spherical particles, with a single particle size injection in different devices ranging from 0.1 to 3 μm, to a mixture of cells with different shapes inherently existing in blood including erythrocytes, leukocytes, and thrombocytes. These components demonstrated identical focusing behavior, further verifying the size-independency of the device with respect to the underlying acoustic forces dominance for capture of cells in defined positions. Furthermore, the high reproducibility of the microchannels, leading to high reproducibility of the resultant focusing of cell mixtures are demonstrated by testing a number of devices (20 devices), all performing at the constant parameters. The consistency in the focusing location as a mean value is shown in Figure 6 C where the extent of the focused cell stream depends only on the cell concentration.

## Materials and Methods

### SAW Device

The technology proposed here, as shown in Figure 3, A and D, is comprised of a standing Rayleigh-type SAW excited upon application of a radiofrequency signal to an opposing pair of λ/4 IDTs on the surface of a double-side polished black LiNbO_3_ 128° *YX* substrate. Once excited at the operating frequency determined by a |S11| minima and first-order estimated by *f* = *C*_*s*_/λ, where *C*_*s*_ represents the propagation velocity of the SAW and λ is the acoustic wavelength equal to the pitch of two consecutive electrodes of same polarity, the displacement generated by one electrode pair will be amplified by constructive interference at the neighboring electrode pairs leading to formation of a high-displacement SAW propagating perpendicular to the electrode fingers. IDTs are composed of 5 nm Ti as adhering layer and 295 nm Al patterned on the substrate using mask-less laser photolithography, electron-beam evaporation of Al, and the lift-off technique. A 100 nm layer of SiO_2_ is then deposited to protect the IDT fingers from corrosion and to promote the adhesion of DFR microchannels to the LiNbO_3_ substrate.

### DFR Microchannels

DFR epoxy polymer used here is DF-3500 owing to the exclusion of antimony, heavy metals and halogens from the material composition, which are among the typical components of currently available DFR materials, making DF-3500 a reliable choice for biological applications. We have thoroughly characterized DF-3500 in terms of the topography and geometry of created structures, using achromatic-confocal optical profilometry (FRT Microprof optical profiler), mechanical profilometry (Bruker Dektak XT), and scanning electron microscopy combined with a focused ion beam analysis technique (SEM-FIB). To optimize the material hardness for the optimum coupling of SAW into the microchannel and obtaining the desired oscillation, structured DF-3500 has been heat treated resulting in E-modulus of 4.02 GPa. The DFR is covalently bound to the piezo-substrate surface through functionalization of a thin layer of SiO_2_ on LiNbO_3_ with epoxy-groups, promoting the strength of the microchannel/LiNbO_3_ bond for high-throughput applications. The microchannel is developed in two stages as demonstrated in Figure 2; first, a sheet of DF-3500 is laminated, using the laminator (Model 305, Fortex Engineering Ltd., UK), then lithographically exposed, using laser-based maskless exposure system (MLA100, Heidelberg Instruments, Germany) to define the microchannel walls which are subsequently developed using cyclohexanone and then heat-treated. The process is repeated for the microchannel cover.

### Experimental Setup

The acoustofluidic chip was placed on a custom chip holder, equipped with a liquid cooling system for constant heat removal from the chip during cell experiments. Human blood samples were obtained from the Deutsches Rotes Kreuz (DRK) Blutspendedienst Nord-Ost gGmbH and diluted using phosphate buffered saline. The blood samples, 3 μm nonfluorescent particles (micromod Partikeltechnologie GmbH), and fluorescent polystyrene particles including 100 nm, 300 nm, and 1 μm (Magsphere Inc.), 200 and 500 nm particle (Polysciences Europe GmbH) were injected to the microfluidic channel using a syringe pump (neMESYS 290N, Cetoni GmbH). The fluidic interconnection consisting of PEEK connectors, metal capillaries, and metal with the chip was achieved by fluid connection blocks with sealing elements, eventually eliminating the need for tubing and significantly decreasing setup time and chip exchange. The electrical connections were developed using spring pins on printed circuit boards, and the SAW was generated using a RF signal source (BelektroniG GmbH). The experiments with fluorescent particles were carried out on the stage of a fluorescence microscope (CellObserver Carl Zeiss AG). For the nonfluorescent ones as well as blood cells, the Leica DMI5000 M (Leica Microsystems GmbH) with Phantom VEO 410 L (Vision Research Inc.) high-speed camera were used.

### Numerical Simulation

The numerical simulations were performed using COMSOL ver 6 on a custom workstation with 32 logical processors at 2.60 GHz operation frequency and 512 GB RAM (see Figure 4). Fully coupled 2D models in the frequency domain were used to simulate acoustic fields for several geometries with varying channel widths and wall thicknesss as well as driving frequencies, whereby the surface normal component of the SAW amplitude was scaled according to measured data by Laser-Doppler vibrometry (UHF 120, Polytec GmbH). To generate SAWs, a harmonic electric potential was applied along rectangular equipotential surfaces, that mimic the pattern of IDT electrodes, on a 128° *YX* LiNbO_3_ substrate domain using a coupled electrostatic module. The coordinate axis of the tensor data set for *z*-normal oriented LiNbO_3_ was rotated 38° around the *x*-axis to accommodate for the particular crystal orientation of the LiNbO_3_ 128° *YX* substrate used experimentally.^52^ Motion of the DFR side walls was obtained by coupling a solid mechanics module. Coupling of acoustic waves into the fluid domain was modeled using the thermoviscous acoustics in the fluid domain acquiring the acoustic pressure and velocity fluctuations. The streaming fields (*i.e., v*_2_) were obtained using the first order solutions to calculate the body force (see eqs 2 and 3 in SI) which is then employed to drive the fluid flow in a laminar flow stationary study.

## Conclusion

In summary we have shown that the laminated DFR microfluidic channels can be integrated onto SAW tweezers, by means of a simple fabrication technique already used in an industrial scale for MEMS, to reliably produce a precisely designed channel dynamic. As such, certain channel dynamics can give rise to the synergetic effect of acoustic forces, *F*_Rad_ and *F*_d–streaming_, thereby resulting in markedly reduced size of the collectable particles. Our DFR-based manufacturing technology, with a few microns resolution on the wafer scale, demonstrates high reproducibility which is crucial in delivering consistent channel dynamics for an effective alignment of acoustic forces equilibrium positions (*F*_Rad_ and *F*_d–streaming_ are minimum) wherein particles are collected. Our SAW tweezer exhibits collection and focusing of spherical particles ranging from 0.2 to 3 μm and biological cells existing in human blood including erythrocytes (7–8 μm), leukocytes (7–20 μm), and thrombocytes (1–4 μm). The excellent ability in collection of particles and cells independent of their dimension and shape, along with reproducibility, cost efficiency, and mass producibility make the integration of DFR onto SAW tweezers promising for translation of SAW microfluidics to real-world medtech implementations.

## Supporting Information Available

Blood cell concentration: Focusing of the human blood components including erythrocytes, leukocytes, and thrombocytes at various concentration (MP4)
Additional details on numerical simulations including material parameters, equations used for calculation of acoustic radiation force and acoustic streaming induced drag force. It also includes further FEA simulation results, particularly showing the acoustic effects in a liquid bound within microchannel with various dimensions (PDF)

## Abbreviations

SAW: surface acoustic wave
BAW: bulk acoustic waves
IDT: interdigital transducer
DFR: dry film resist
PDMS: polydimethylsiloxane
*F*_Rad_: acoustic radiation force
*F*_d-streaming_: acoustic streaming induced drag force
LiNbO_3_: lithium niobate substrate
Al: aluminum
MEMS: microelectromechanical systems
SiO_2_: silicon dioxide
FEA: finite element analysis
