# Supplementary material for: Fully Microfabricated Surface Acoustic Wave Tweezer for Collection of Submicron Particles and Human Blood Cells
Source: ACS Appl Mater Interfaces. 2023 May 15;15(20):24023–33. doi: 10.1021/acsami.3c00537 (PMC10215297; doi:10.1021/acsami.3c00537)
Supplement: Supplementary file 2 — am3c00537_si_002.pdf [file am3c00537_si_002.pdf]

Supporting Information

Fully microfabricated surface acoustic wave tweezer for collection of submicron particles and human blood cells

*Armaghan Fakhfouri * † Melanie Colditz,† Citsabehsan Devendran,‡ Kateryna Ivanova,† Stefan Jacob,⁋ Adrian Neild,‡ Andreas Winkler*†*

†Leibniz-IFW Dresden, Helmholtzstr. 20, 01069 Dresden, Germany.

‡Department of Mechanical and Aerospace Engineering Monash University, Clayton, Victoria 3800, Australia

⁋Physikalisch-Technische Bundesanstalt, Bundesallee 100, 38116, Brunswick, Germany

*Corresponding authors: E-mail: a.fakhfouri@ifw-dresden.de; a.winkler@ifw-dresden.de;

Numerical Simulations

Making use of DFR for acoustofluidic manipulation, to the best of our knowledge, has not been reported so far. Here, we have first optimized the fabrication parameters, for instance development and heat-treatment, to maximize the desired SAW-DFR coupling. The material was then characterized and the evaluated properties were used for the FEA simulation, as shown in Table. 1. Here, we propose a 2-dimensional model capturing the acoustic effects in the microchannel cross-sectional plane, considering the uniformity of such effects along the channels' length. Figure S2 indicate the acoustic pressure field distribution, acoustic streaming field and the resultant acoustic forces including radiation force (red arrows) and streaming induced drag force (surface plot). The final location of particles in such systems, as shown in Fig. 1 E-G, is determined according to the aligned equilibrium position of both forces.

Table S1. Properties of DF-3500 material

Density	ρ_{DF}	1233 kg m^{-3}
Young's modulus	E_{DF}	4.1 GPa
Poisson's ratio	v_{DF}	0.22

Table S2. Properties of Lithium Niobate (128˚ YX-cut $LiNbO_3$) at T = 25 ˚C

Density	ρ_{LN}	4700 kg m^{-3}
Speed of sound	c_{LN}	3994 m s^{-1}

Table S3. Properties of water at T = 25 ˚C

Density[1]	ρ_0	997 kg m^{-3}
Speed of sound[1]	c_0	1497 m s^{-1}
Shear viscosity[1]	η	0.890 mPa s
Bulk viscosity[2]	η′	2.47 mPa s
Compressibility[2]	κ_0	448 TPa^{-1}
Thermal conductivity[2]	k_{th}	0.603 W m−1 K^{-1}
Specific heat capacity[2]	C_P	4183 J kg−1 K^{-1}
Thermal expansion coefficient[2]	α_{th}	2.97 × 10−4 K^{-1}

Calculation of acoustic radiation force

The time-averaged absolute acoustic radiation pressure field ($\langle|P_1|\rangle$ was used to numerically calculate acoustic radiation force employing Equation. 1 [3].

$$\boldsymbol{F}_{rad} = -\pi r^3 \left[\frac{2\kappa_0}{3} Re[f_1^* P_1^* \nabla P_1] - \rho_0 Re[f_2^* \boldsymbol{v}_1^* \nabla \boldsymbol{v}_1]\right] \tag{1}$$

where $$f_1 = 1 - \frac{\kappa_P}{\kappa_0} ,$$

$$f_2 = \frac{2(1-\gamma)(\rho_P - \rho_0)}{2\rho_P + \rho_0 (1-3\gamma)} ,$$

,

$$\gamma = -\frac{3}{2}[1 + i(1 + \delta_v)]\delta_v, \quad ,$$

$$\delta_v = \frac{\sqrt{2\eta}}{r\sqrt{\omega\rho_0}}$$

P_1 and $\boldsymbol{v}_1$ are the first order pressure and velocity and ρ_P and ρ_0 represent the density of particle and fluid, respectively. κ_P and κ_0 are compressibility of particle and fluid. η represents dynamic viscosity coefficient, ω is the angular frequency and * means the complex conjugated.

Calculation of acoustic streaming induced drag force

The driving force of the acoustic streaming, namely, second-order steady state velocity field $\boldsymbol{v}_2$, was evaluated based on the first-order pressure (P_1) and velocity ($\boldsymbol{v}_1$) fields using Reynolds stress[4]

.

$$\langle \boldsymbol{F} \rangle = \rho_0 \langle (\boldsymbol{v}_1 . \boldsymbol{\nabla}) \boldsymbol{v}_1 + \boldsymbol{v}_1 \boldsymbol{\nabla} . \boldsymbol{v}_1 \rangle \tag{2}$$

$$\langle \boldsymbol{F} \rangle = -\boldsymbol{\nabla} P_{\boldsymbol{2}} + \left[\eta' + \left(\frac{4}{3} \right) \eta \right] \boldsymbol{\nabla} (\boldsymbol{\nabla} . \boldsymbol{v}_2) + \eta \boldsymbol{\nabla}^2 \boldsymbol{v}_2 \tag{3}$$

Acoustic streaming induces drag force on the immersed particles with an initial velocity of $\boldsymbol{v}_P$, according to the equation 4 [2]

$$\boldsymbol{F}_{\text{drag}} = 6\pi \eta r (\boldsymbol{v}_2 - \boldsymbol{v}_P) \tag{4}$$

where η and η′ indicate the dynamic viscosity coefficient and bulk viscosity coefficient, respectively. P_2 represents the second order pressure field.

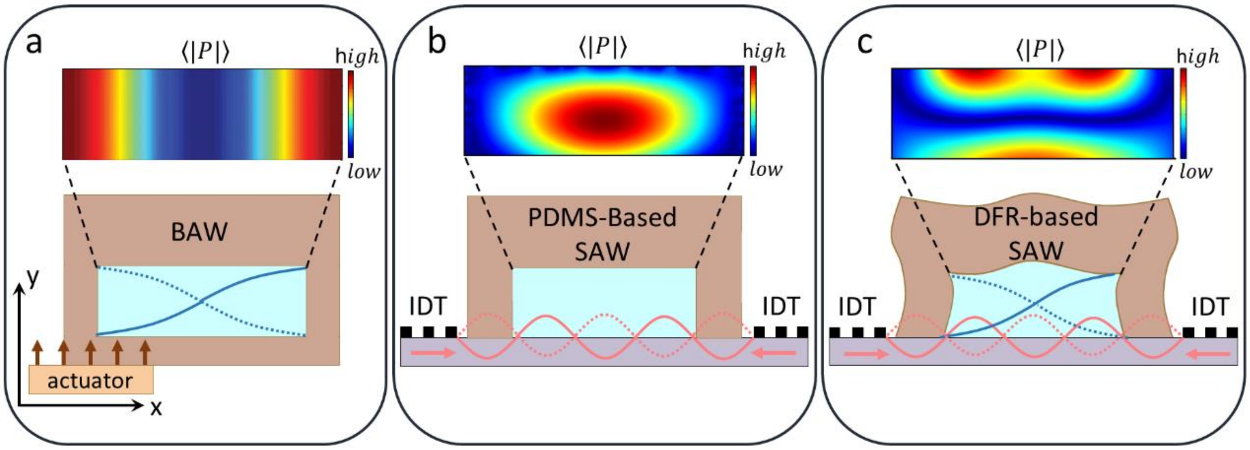

Figure S1. The conventional and proposed acoustofluidic technologies together with the FEM-simulated time-averaged absolute pressure field across the channel width: (a) bulk acoustic waves (BAW) excited across the fluid volume, (b) standing surface acoustic wave excitation (SSAW) of an acoustically soft channel, i.e. polydimethylsiloxane (PDMS), and (c) SSAW-induced higher-mode resonance in a DFR microchannel; all simulations of time-averaged pressure field were done for the same channel dimensions.

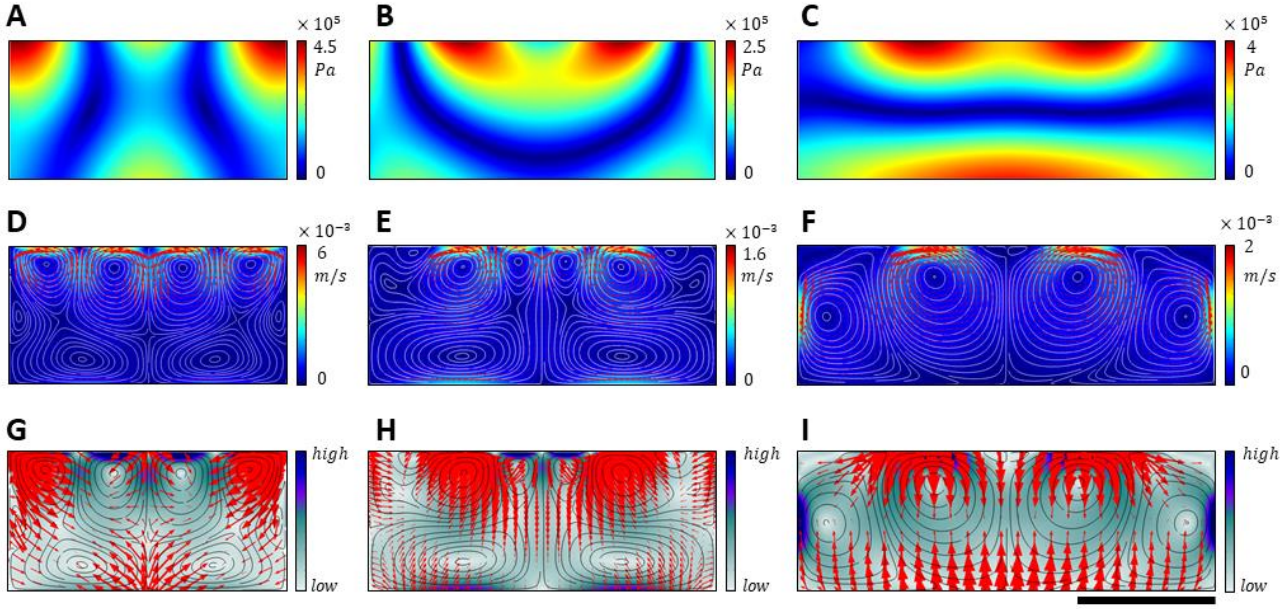

Figure S2. FEA numerical simulation of acoustic pressure and acoustic streaming fields along with the resultant forces. (A-C) Surface plot of the time-averaged absolute pressure field ($\langle|P_1|\rangle$), (D-F) Streamlines illustrating the simulated streaming field ($v_2 2$) and (G-I) Resultant acoustic forces including acoustic radiation force, F_{Rad}, illustrated with red arrows, and streaming induced drag force field, $F_{d\text{-streaming}}$, shown in surface plots, where two opposing SAW with 300μm wavelength (λ_{SAW}) = 300 μm) are coupled to $\lambda_{SAW}/6$ high channels with a width of (A & D & G) $\lambda_{SAW}/3$, (B & E & H) $\lambda_{SAW}/2.5$ and, (C & F & I) $\lambda_{SAW}/2$. The simulated displacement field is adjusted to wavefield measurement results by laser doppler vibrometry reading 0 to 5×10^{-4} μm. Scale bar equals $\lambda_{SAW}/6$.

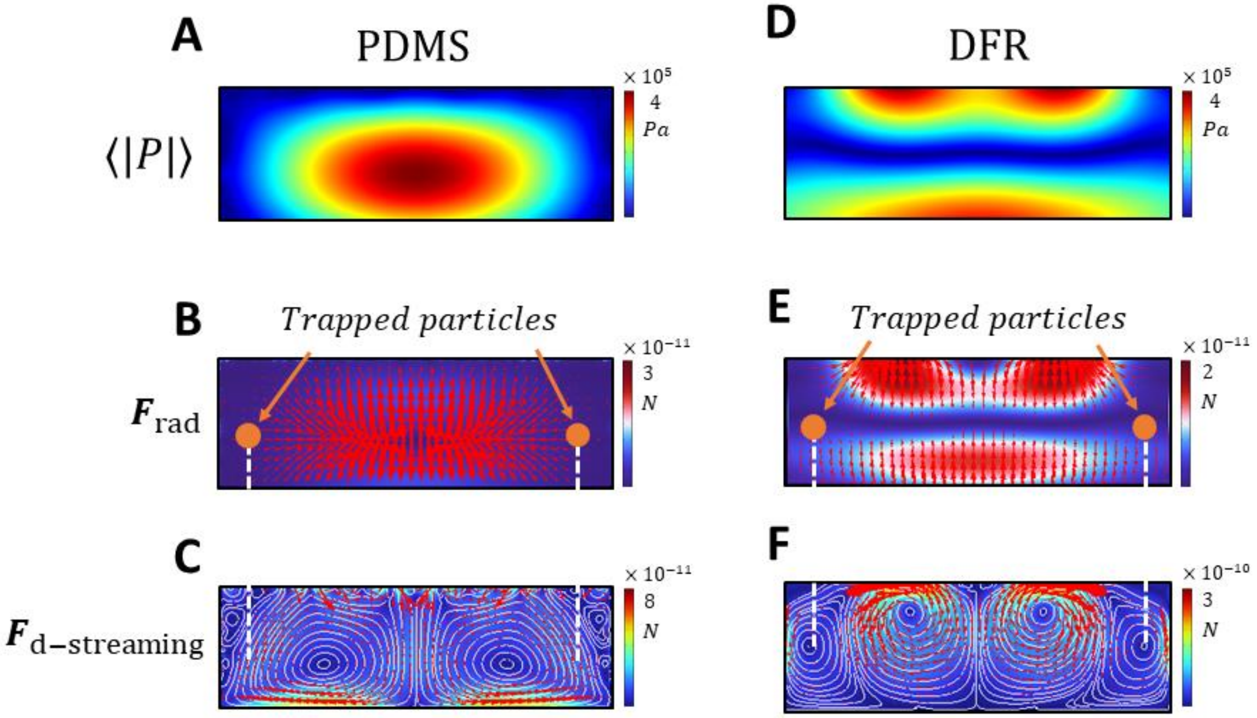

Figure S3. (A & D) Time averaged acoustic pressure field $\langle|P_1|\rangle$, along with (B & E) Acoustic radiation force (F_{rad}) and (C & F) acoustic streaming induced drag force ($F_{d\text{-}streaming}$) for 1 μm particle in a liquid bound by (λ_{SAW}/6)-high, (λ_{SAW}/2)-wide (A-C) Polydimethylsiloxan (PDMS) and (D-F) dry-film-resist (DFR). The simulated displacement field is adjusted to wavefield measurement results by laser doppler vibrometry reading 0 to 5×10^{-4} μm.

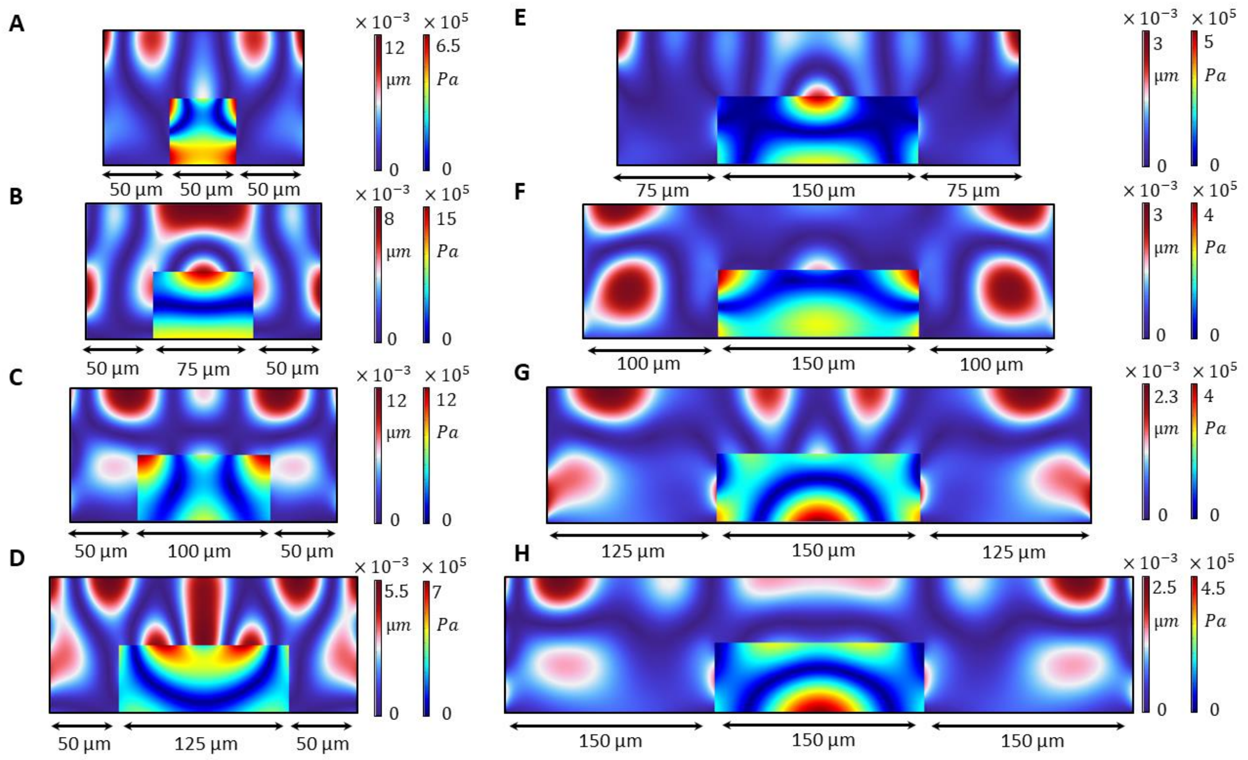

Figure S4. FEA numerical simulation of channel cross section steady state displacement and acoustic pressure field ⟨|P_1|⟩, in 50-µm-high channels with constant cover thickness of 50 µm but (A-D) different width and (E-H) different wall thickness. The simulated displacement field is adjusted to wavefield measurement results by laser doppler vibrometry reading 0 to 5 $\times 10^{-4}$ µm.

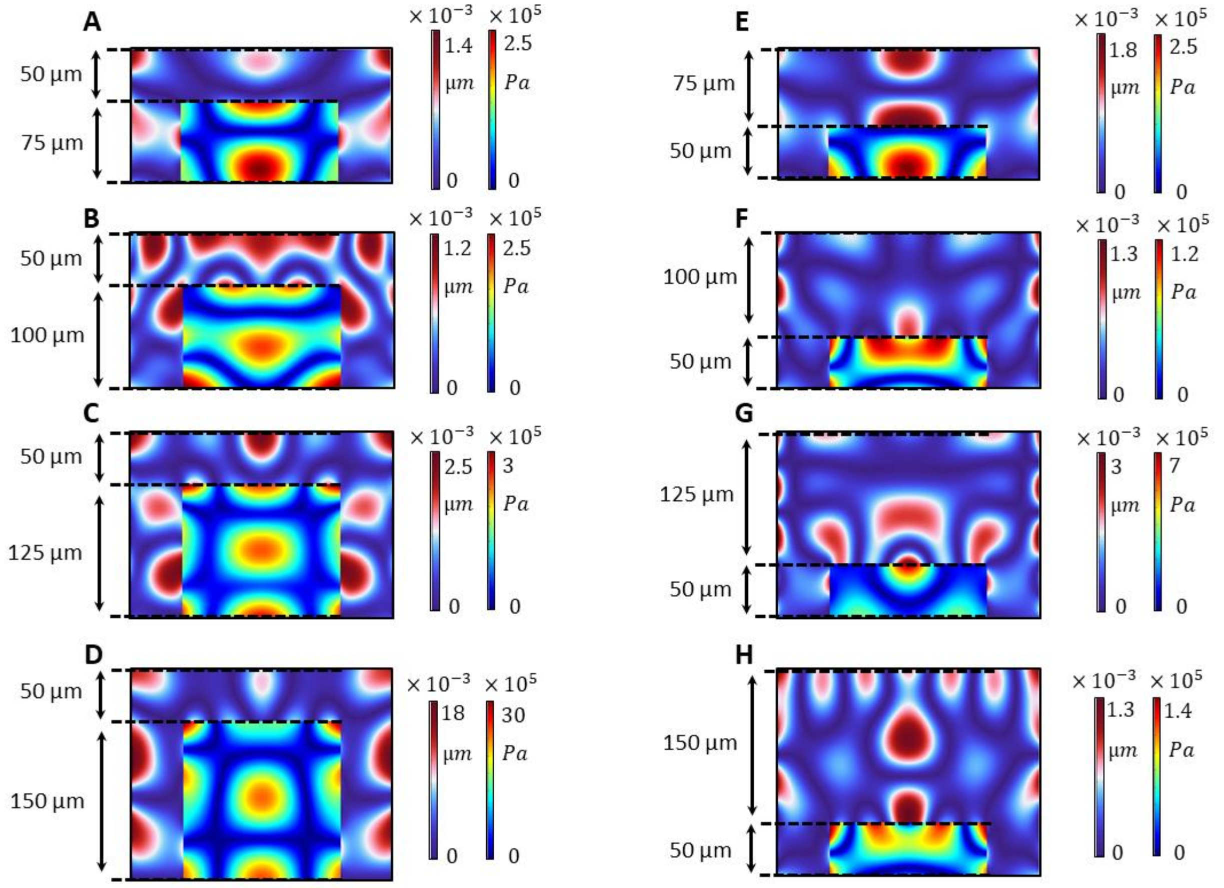

Figure S5. FEA numerical simulation of channel cross section steady state displacement and acoustic pressure field $\langle |P_1| \rangle$, in 50-µm-wide channels with constant wall thickness of 50 µm but (A-D) different channel height and (E-H) different cover thickness. The simulated displacement field is adjusted to wavefield measurement results by laser doppler vibrometry reading 0 to 5×10^{-4} µm.

Movie. S1. Focusing of the human blood components. Cell mixture includes erythrocytes, leukocytes, and thrombocytes, flowing at 25 µl/min. The SAW field operates at 12.8 MHz and applied power is 400 mW.

References

1. Haynes, W. M., CRC Handbook of Chemistry and Physics. *CRC press:* 2016.
2. Muller, P. B.; Barnkob, R.; Jensen, M. J.; Bruus, H., A Numerical Study of Microparticle Acoustophoresis driven by Acoustic Radiation Forces and Streaming-induced Drag Forces. *Lab Chip* **2012,** *12* (22), 4617-27.
3. Settnes, M.; Bruus, H., Forces Acting on a Small Particle in an Acoustical Field in a Viscous Fluid. *Physical Review E* **2012,** *85* (1), 016327.
4. Devendran, C.; Albrecht, T.; Brenker, J.; Alan, T.; Neild, A., The Importance of Travelling Wave Components in Standing Surface Acoustic Wave (SSAW) Systems. *Lab on a Chip* **2016,** *16* (19), 3756-3766.
